# Age, Education Years, and Biochemical Factors Are Associated with Selective Neuronal Changes in the Elderly Hippocampus

**DOI:** 10.3390/cells11244033

**Published:** 2022-12-13

**Authors:** Carla Cristina Miranda Castro, Sayonara Pereira Silva, Lívia Nascimento Rabelo, José Pablo Gonçalves Queiroz, Laura Damasceno Campos, Larissa Camila Silva, Felipe Porto Fiuza

**Affiliations:** Graduate Program in Neuroengineering, Edmond and Lily Safra International Institute of Neuroscience, Santos Dumont Institute, Macaíba 59280-000, Brazil

**Keywords:** vesicular glutamate transporter 1, GABA transporter 1, SLC17A7, SLC6A1, inflammaging

## Abstract

Brain aging involves regional alterations of specific cellular subpopulations in the human hippocampus: a network hub for memory consolidation. The present study investigates whether age, sex, education years, and the concentration of neuropathological and inflammatory proteins influence neuronal-type marker expression in the elderly hippocampus. We analyzed the digital images (1 µm/pixel) of postmortem hippocampal sections from 19 non-demented individuals (from 78 to 99 years). This material was obtained from the “Aging Dementia and TBI Study” open database. Brain samples were processed through in situ hybridization (ISH) for the immunodetection of VGLUT1 (glutamatergic transporter) and GAT1 (GABAergic transporter) and mRNAs and Luminex protein quantifications. After image acquisition, we delineated the dentate gyrus, CA 3/2, and CA1 hippocampal subdivisions. Then, we estimated the area fraction in which the ISH markers were expressed. Increased VGLUT1 was observed in multiple hippocampal subfields at late ages. This glutamatergic marker is positively correlated with beta-amyloid and tau proteins and negatively correlated with interleukin-7 levels. Additionally, education years are positively correlated with GAT1 in the hippocampus of elderly women. This GABAergic marker expression is associated with interferon-gamma and brain-derived neurotrophic factor levels. These associations can help to explain how hippocampal sub-regions and neurotransmitter systems undergo distinct physiological changes during normal aging.

## 1. Introduction

Aging is the primary risk factor for conditions such as cancer, cardiovascular diseases, and neurodegeneration [1,2]. Such involvement with a broad range of life-threatening processes occurs because aging affects all organ systems [3]. However, age-related physiological deterioration develops at different rates across the multiple biological tissues of an individual [4,5].

In brain aging, neuronal reductions are found in restricted portions of the prefrontal, diencephalic, brainstem, cerebellar, and hippocampal areas, while other brain regions are relatively spared [6,7,8,9,10,11]. This process is also cell-type specific since neuronal subpopulations are differentially impacted by aging [12,13,14,15]. Many factors, such as sex differences, lifestyle habits, or “inflammaging”, could explain why some brain regions and cell types are more likely to be affected by aging [16,17,18].

The hippocampus is a key component of the brain’s limbic system and undergoes age-related structural changes that are linked to cognitive decline [19]. This region is involved in the consolidation of declarative memories and context-dependent spatial learning [20,21]. Anatomically, the hippocampal formation is subdivided into the dentate gyrus (DG), the 1–4 fields of Cornu Ammonis (CA), and the subiculum [22,23]. The aged hippocampus is characterized by a global reduction in volume [24], but the total number of neurons are lost only in the DG and subiculum [6]. Although detailed transcriptional profiles are available for aged non-human primates, little is known regarding how cellular subpopulations are differentially affected by aging in the human hippocampus [25].

The hippocampal cellular microcircuits are composed of two major neuronal classes: excitatory glutamatergic cells and inhibitory GABAergic interneurons [26]. Glutamate is the most abundant neurotransmitter in the central nervous system. In the presynaptic neuron, glutamate is stored in vesicles by vesicular glutamate transporters (VGLUTS) types 1–3 [27]. While VGLUT1 is preferentially expressed in the hippocampus, cerebral cortex, and cerebellum, VGLUT2 is found mainly in the subcortical regions, and VGLUT3 occurs scarcely through the brain [28]. In animal studies, VGLUT mRNA and protein levels can be found to be increased or decreased during aging, depending on the brain region [29,30]. In humans, a considerable body of evidence points to an age-related reduction in glutamate levels [31,32]. However, there is little data regarding VGLUT changes, especially in nonagenarians and centenarians [33].

About 10–20% of all neurons in the hippocampus release the inhibitory neurotransmitter GABA [34]. GABA is synthesized by glutamic acid decarboxylase (GAD) and is removed from the synaptic cleft by GABA transporters (GATs) types 1–3 [35]. It has been previously shown how aging affects the expression of multiple GABAergic markers in the human cerebral cortex, including GAD isoforms and GAT1 [36]. This topic was also investigated in the human hippocampus, but data regarding GAT1 expression is still unavailable [37].

The present study characterizes how VGLUT1 and GAT1 mRNA expression changes in the hippocampal subfields of non-demented individuals within the octogenarian-nonagenarian age range. It also describes how biological sex, education years, and the local concentration of neuropathological, inflammatory, and neurotrophic mediators are associated with this expression. We found a relationship between the glutamatergic marker expression, age, and local levels of inflammatory mediators and neuropathological proteins. The GABAergic marker was associated with education years in females as well as the concentrations of neurotrophic and inflammatory proteins. The identification of these associations might help to explain why some individuals are less susceptible to reaching old age and present with cognitive decline.

## 2. Materials and Methods

### 2.1. Brain Samples

High-resolution digital images of postmortem human brains from 19 donors (9 males and 10 females, ranging from 78 to 99 years) were obtained from the Aging, Dementia and Traumatic Brain Injury Study, an open database compiled by the University of Washington, the Kaiser Permanente Washington Health Research Institute, and the Allen Institute for Brain Science, (http://aging.brain-map.org/, accessed on 14 October 2022). The database presents neuropathological, molecular, and transcriptomic data from 107 participants of the Adult Changes in Thought (ACT) study: a prospective cohort focusing on randomly selected and aged individuals from the Seattle—USA area [38]. Major findings with this dataset were previously reported by [39]. All permissions for data usage in the academic research and derived publications are explicit at the following links of the database: https://alleninstitute.org/legal/terms-use/ and http://aging.brain-map.org/overview/home (accessed on 14 October 2022). For referencing histological images and other datasets, we followed the citation guidelines available at https://alleninstitute.org/legal/citation-policy/ (accessed on 14 October 2022).

Educational attainment was obtained from the self-reports of the participants enrolled in the ACT. The participants also received follow-up visits every 2 years in which they were administered the Cognitive Abilities Screening Instrument [40]. The results of these evaluations and clinical data were reviewed to attribute a DSM-IV dementia diagnosis. This diagnosis was confirmed by the neuropathological evaluation (Braak, CERAD, and NIA-Reagan scores) of brain samples. All participants consented to the donation of brain samples at the autopsy.

### 2.2. Tissue Processing

We will briefly summarize the histological steps that brain samples were submitted to, which were performed at the Allen Institute (ISH) or at the University of Washington (Luminex). These procedures are described in detail at the following link: https://help.brain-map.org/display/aging/Documentation (accessed on 14 October 2022). All donors were submitted to a rapid autopsy protocol after a postmortem interval of <8 h. This procedure involves the collection of ventricular cerebrospinal fluid and mid-sagittal hemisections from 60 brain tissue samples. These samples were flash frozen in liquid nitrogen and stored at −80 °C. All remaining tissue was immersion-fixed in 10% normal buffered formalin for 2–3 weeks. Routine diagnostics stains, such as hematoxylin-eosin and luxol fast blue, were used for the neuropathological evaluation of tissue viability.

Hippocampal tissue was sectioned at 25 µm thickness in a Leica CM3050S cryostat (Leica Biosystems, Deer Park, TX, USA). Hippocampal sections were collected in a systematic and uniform interval as a prerequisite for stereological sampling. In the first two sections, RNA quality was assessed to ensure the suitability of the postmortem samples for molecular and morphological analysis. RIN quality was determined using MELT Total Nucleic Acid Isolation System AM1983 (Ambion, Austin, TX, USA). Each section was collected and homogenized into 100 μL of a MELT buffer/cocktail mix for RNA isolation. The isolated RNA was standardized to a concentration of 5 ng/μL and 1.0 μL and was run on a Pico chip using the Bioanalyzer 2100 (Agilent, Santa Clara, CA, USA). Finally, RNA integrity numbers (RIN) ranging from 1 to 10 were generated [41]. Every sample included in the present study was above RIN 5, in accordance with previous studies [42,43].

A standard colorimetric ISH protocol [44] was employed to label the target mRNAs which originated from the expression of genes that were considered to be canonical cell markers. Three hippocampal sections were collected in an interval of 20 for each ISH marker assay. In this design, sections 8, 28, and 48 were processed for the solute carrier family 17, member 7 (SLC17A7), and sections 10, 30, and 50 were processed for solute carrier family 6, member 1 (SLC6A1). SLC17A7 is another nomenclature for VGLUT1. Similarly, SLC6A1 is another nomenclature for GAT1. Hereafter, we preferentially refer to these markers as VGLUT1 or GAT1. In addition, seven hippocampal sections (1/10 interval) were collected for Nissl staining in 0.213% thionin.

For ISH procedures, slides were fixed for 20 min in 4% neutral buffered paraformaldehyde (PFA), rinsed in 1× PBS, were acetylated for 10 min in 0.1M triethanolamine with 0.25% acetic anhydride, and dehydrated using a graded series of 50%, 70%, 95%, and 100% ethanol. The first steps blocked endogenous peroxidase activity and permeabilized the tissue, followed by the subsequent hybridization of digoxigenin-labeled probes to target mRNA. After a series of washes to eliminate the excess probe, the slides were incubated with horseradish peroxidase (HRP)-conjugated anti-digoxigenin antibody and a biotin-coupled tyramide. This step originated a blue/purple precipitate, revealing the remaining probe in the tissue. ISH controls were addressed with the inclusion of the human gene probe GAP43 to gauge a signal from a probe with typically high expression, and CALB1 was used to gauge the signal of a probe with a typically moderate expression. Finally, high-resolution digital images of the coronal brain sections were acquired using the Leica ScanScope automated scanner (Leica Biosystems). The slices were scanned with 20× objectives (0.75 NA Pan Apo), and images were captured at cellular resolution (1 µm/pixel). For each donor, protein quantification data and Nissl/ISH images can be freely downloaded at http://aging.brain-map.org/donors/summary (accessed on 14 October 2022).

The local concentrations of proteins involved in neuropathological, inflammatory, and neurotrophic events were determined through a multiplexed Luminex assay (Table 1). Frozen hippocampal tissue, immediately adjacent to those submitted for ISH, was sequentially homogenized and centrifuged in a RAB buffer, followed by 5M guanidine-HCL or a RIPA buffer. This yielded supernatant-labeled RAB extracts, G extracts, or RIPA extracts, respectively. These extracts were incubated with detector antibodies, and fluorescence was analyzed to determine the sample concentration from the standard curve. This was performed in a LiquiChip Workstation (Qiagen). RAB extracts were utilized to quantify brain-derived neurotrophic factor (BDNF) and 11 plex proteins, G extracts were used to quantify Amyloid beta (Ab) 40 and 42 isoforms, and the Tau protein and Tau isoform abnormally phosphorylated at threonine 181 (pTau-181). RIPA extracts were used to quantify alpha-synuclein.

### 2.3. Case Eligibility

**Figure A1:**
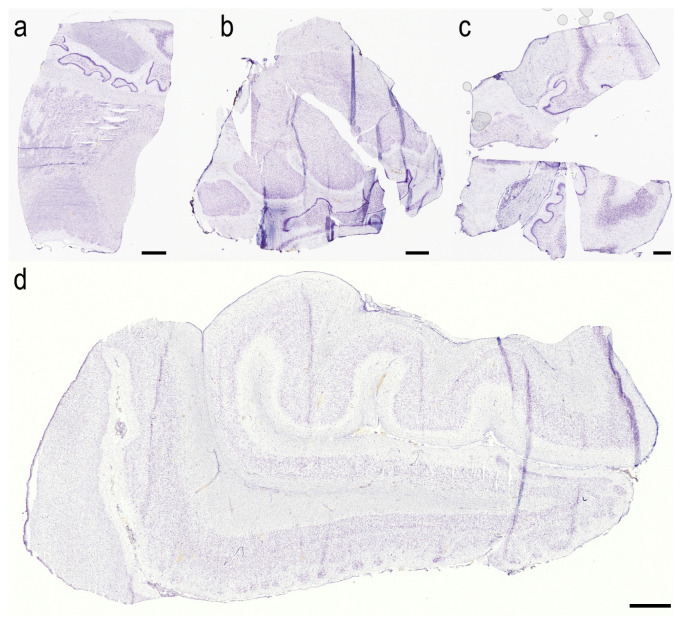
Examples of excluded samples. Material from non-demented donors were excluded if the hippocampal regions were not available due to distinct anatomical level or damages from the histological processing. This is illustrated by the samples from donors H14.09.081 (**a**), H14.09.028 (**b**), H14.09.014 (**c**), and H14.09.076 (**d**). Images were obtained from the Aging, Dementia and TBI study database and are publicly available at: http://aging.brain-map.org/ (accessed on 14 October 2022). Anatomical charts were produced by the authors of the present work. Scale bars 2000 µm.

For the present study, we first performed a screening within the specimens obtained from all 107 donors available in the database. To pass our eligibility criteria, specimens had to: (1) Originate from individuals without TBI (54 out of 107); (2) Originate from individuals with “No Dementia” as DSM-IV diagnosis (30 out of 54); (3) Be hippocampal sections at an uncus-lateral geniculate level with dentate gyrus and CA regions identifiable (19 out of 30). This yielded hippocampal samples from 19 non-TBI and non-demented donors for analysis (Table 2). Examples of excluded cases can be found in the Appendix A (Figure A1). Non-TBI donors diagnosed with dementia were not included in this study. Among the samples available for this group, only a small number of hippocampal tissues were preserved with intact anatomical features (a total of 7 samples).

### 2.4. Image Analysis

**Figure 1:**
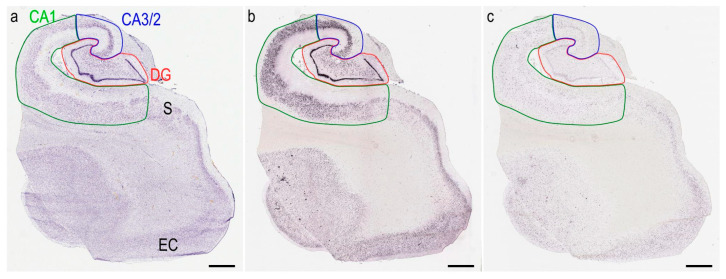
Images from hippocampal coronal sections processed through the Nissl method (**a**) and in situ hybridization for VGLUT1 (**b**) or GAT1 (**c**). This material was obtained from the donor H14.09.074. Nissl images were used as a template for the identification of hippocampal subfields. The dentate gyrus (DG, red), cornu ammonis 3/2 (CA3/2, blue) and 1 (CA1, green) were drawn for cell quantification. Identification of the subiculum (S) and entorhinal cortex (EC) was not possible for all samples. Images were obtained from the Aging, Dementia and TBI study database and are publicly available at: http://aging.brain-map.org/ (accessed on 14 October 2022). Anatomical charts were produced by the authors of the present work. Scale bars 2000 µm.

**Figure 2:**
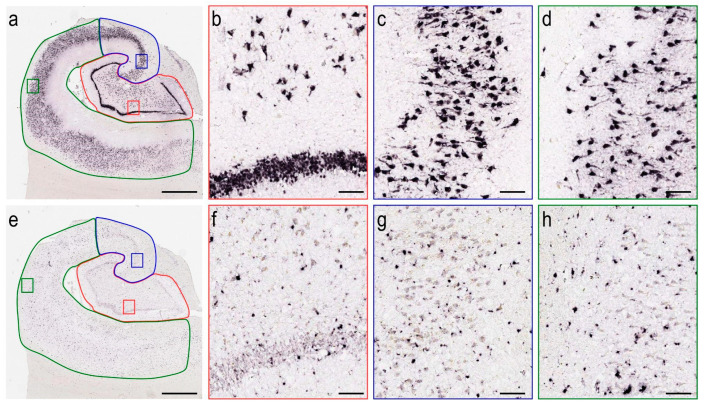
VGLUT1 and GAT1 mRNA immunoreactive profiles in the hippocampal sub-regions. This material was obtained from the donor H14.09.074. VGLUT1 (**a**) positive cells are observed at high-resolution in the DG (red, (**b**)), CA3/2 (blue, (**c**)), and CA1 (green, (**d**)). GAT1 (**e**) positive cells are observed at high-resolution in the DG (red, (**f**)), CA3/2 (blue, (**g**)), and CA1 (green, (**h**)). Images were obtained from the Aging, Dementia and TBI study database and are publicly available at: http://aging.brain-map.org/ (accessed on 14 October 2022). Anatomical charts were produced by the authors of the present work. Scale bars 2000 µm (**a**,**e**) and 200 µm (**b**–**d**,**f**–**h**).

We downloaded full-resolution VGLUT1 and GAT1 images from the database. Prior to morphometric analyses, we delimited the hippocampal subfields of the dentate gyrus, CA 3/2, and CA 1. These regions were delimited following the anatomical criteria described in previously published papers [6,45,46]. To ensure the accuracy of the region delimitation, we overlaid the ISH images with the corresponding Nissl sections using the software FIJI ImageJ version 1.52p (National Institutes of Health, Bethesda, MD, USA). We matched anatomical landmarks (e.g., blood vessels) to accurately fit every ISH-Nissl image pair upon each other. Thus, we used Nissl cytoarchitectonic features as a template for the region delimitation in the ISH images. We traced the DG boundaries, including the molecular layer, granule cell layer, and hippocampal hilus. The CA3/2 boundaries were determined upon the identification of tightly packed cell bodies of large pyramidal neurons. Given the cell density in this area, it was not possible to trace a clear border between CA 2 and CA 3. The CA 1 limits were traced to encompass the more radially dispersed pyramidal neurons (Figure 1). A relevant number of the available samples did not present the Subiculum, and therefore, this region was not included in this study. VGLUT1 and GAT1 mRNA immunosignals were identified in all hippocampal regions by the dark immunoreactive reactions within the histological material (Figure 2).

The percentage of the area (area fraction) occupied by VGLUT1 and GAT1 staining was estimated through the area fraction fractionator stereological methodology [47]. This was performed in a built-in function of the software StereoInvestigator v11.0 (MBF Bioscience, Williston, ND, USA). After region delimitation, the software superimposed a Cavalieri point-counting grid in the blind-coded ISH image. We employed a 500 µm × 500 µm XY grid with a 25 µm distance between each point. In this manner, each grid point was associated with an area of 625 µm^2^. Two independent observers checked every hippocampal subfield for hit points superimposed on ISH signals. The hit points are correlated with the area occupied by the ISH markers, whereas the total number of points indicates the total regional area. Thus, the percentage of hit points per total point indicates the area fraction occupied by the ISH marker. In addition to the regional estimations, we also used a single delimitation encompassing the DG, CA3/2, and CA1 to estimate the ISH area fraction for the total hippocampus.

### 2.5. Statistics

We confirmed the normality of our group distributions after Kolmogorov–Smirnov tests and evaluation of kurtosis and skewness. We first evaluated whether the VGLUT1 and GAT1 area factions differed between the sex and age groups with an unpaired Student *t*-test. Since the database lists individuals over 90 years in categorical intervals of age (90–94 or 95–99), we could not assess Pearson’s correlation with this variable. Instead, we divided the age group into 78–89 years (n = 10) and 90–99 years (n = 9). Education years, however, are presented as a continuous variable. We performed Pearson’s correlation of the ISH marker area fraction with education years in the total pool of the samples, as well as in donors stratified by sex. Lastly, we performed Pearson’s correlation of the ISH marker area fraction with neuropathological and inflammatory protein quantifications obtained through Luminex. Descriptive results are displayed as the mean ± standard deviation, and *p*-values were set with α < 0.05. All statistical analyses were performed with the software GraphPad Prism version 7.0 (GraphPad, San Diego, CA, USA). The Pearson correlation matrix was constructed using Rstudio software (Rstudio team, Boston, MA, USA).

## 3. Results

### 3.1. Effects of Age and Sex on Hippocampal Neuron-Specific Markers

**Figure 3:**
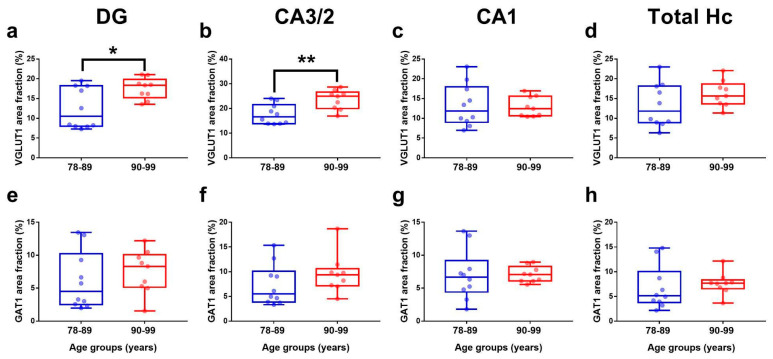
VGLUT1 (**a**–**d**) and GAT1 (**e**–**h**) area fractions in the hippocampus from non-demented donors grouped by age. Donors were divided into 78–89 (blue) or 90–99 (red) year-old groups. Student *t*-tests between these groups were performed in the DG, CA3/2, CA1, and total hippocampus (Hc). Data are presented as boxplots with lines at the mean. * *p*-values < 0.05, ** *p*-values < 0.01.

The VGLUT1 and GAT1 hippocampal area fractions were compared between 78–89 and 90–99-year-old groups. Samples from the elderly above 90 years presented a higher percentage of the area occupied by the VGLUT1 ISH signal in the DG (t (17) = 2.60; *p* = 0.019; Figure 3a) and CA 3/2 (t (17) = 3.25; *p* = 0.005; Figure 3b). However, this was not observed in CA 1 (t (17) = 0.20; *p* = 0.84; Figure 3c) nor in the total hippocampus (t (17) = 1.39; *p* = 0.18; Figure 3d). The analysis for the GAT1 area fraction did not reveal any differences between these age groups in DG (t (17) = 0.46; *p* = 0.74; Figure 3e), CA3/2 (t (17) = 1.21; *p* = 0.24; Figure 3f), CA1 (t (17) = 0.05; *p* = 0.96; Figure 3g), or in the total hippocampus (t (17) = 0.52; *p* = 0.60; Figure 3h).

**Figure 4:**
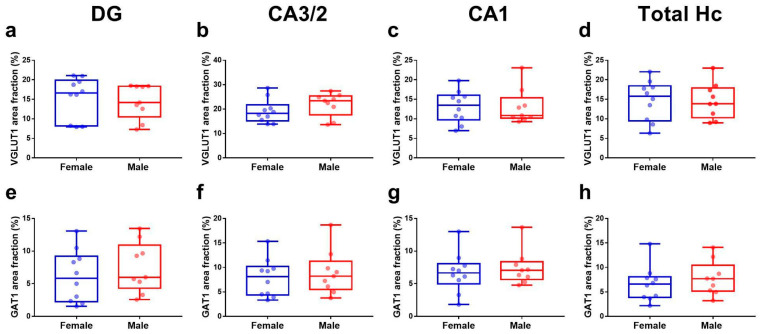
VGLUT1 (**a**–**d**) and GAT1 (**e**–**h**) area fractions in the hippocampus from non-demented donors grouped by sex. Donors were divided by female (blue), male (red), and year-old groups. Student *t*-tests between these groups were performed in the DG, CA3/2, CA1, and total hippocampus (Hc). Data are presented as boxplots with lines at the mean. *p*-values < 0.05 were considered significant.

The VGLUT1 and GAT1 hippocampal area fractions were also compared between the male and female groups. No sex differences were found between the VGLUT1 area fraction in DG (t (17) = 0.45; *p* = 0.65; Figure 4a), CA3/2 (t (17) = 1.23; *p* = 0.23; Figure 4b), CA1 (t (17) = 0.004; *p* = 0.99; Figure 4c), or in the total hippocampus (t (17) = 0.04; *p* = 0.96; Figure 4d). Similarly, no sex differences were observed between the GAT1 area fractions in DG (t (17) = 0.76; *p* = 0.45; Figure 4e), CA3/2 (t (17) = 0.56; *p* = 0.58; Figure 4f), CA1 (t (17) = 0.57; *p* = 0.58; Figure 4g), or in the total hippocampus (t (17) = 0.72; *p* = 0.48; Figure 4h).

### 3.2. Effects of Education Years on Hippocampal Neuron-Specific Markers

**Figure 5:**
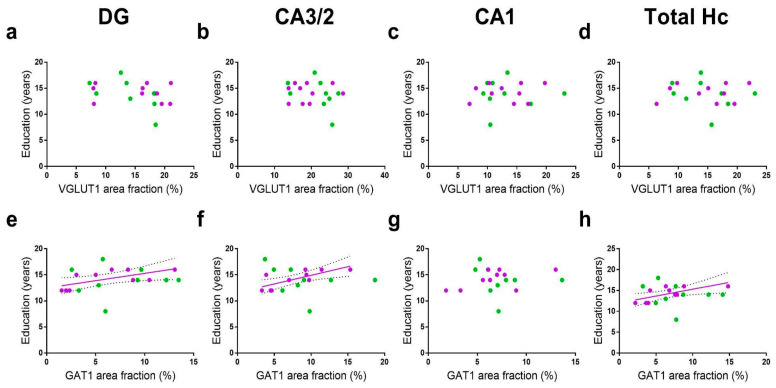
Pearson’s correlations between VGLUT1 (**a**–**d**) and GAT1 (**e**–**h**) area fractions with education years. Analysis was performed in the total pool of donors as well as donors divided in female (purple) or male (green) groups. Area fractions are plotted as a function of education years in the DG, CA3/2, CA1, and total hippocampus (Hc). Regression lines with 95% confidence intervals are shown when a significant (*p* < 0.05) relationship is observed.

We evaluated whether VGLUT1 and GAT1 occupied areas were correlated with the education years of the donors. Analyzing the total pool of the subjects, no correlations between these variables were found for any ISH marker on any hippocampal subdivision (Table A1). However, when donors were stratified by sex, the female samples presented positive correlations between the education years and GAT1 area fractions in the DG (r = 0.66, r^2^= 0. 44, *p* = 0.036), CA 3/2 (r = 0.76, r^2^= 0. 56, *p* = 0.011), and in the total Hc (r = 0.70, r^2^= 0. 49, *p* = 0.024). This correlation was not significant in the CA1 region (r = 0.56, r^2^= 0. 31, *p* = 0.09). No significant correlations in the education years and VGLUT1 or GAT1 area fractions were found in the male samples (Figure 5).

### 3.3. Associations of Biochemical Factors with Hippocampal Neuron-Specific Markers

**Figure 6:**
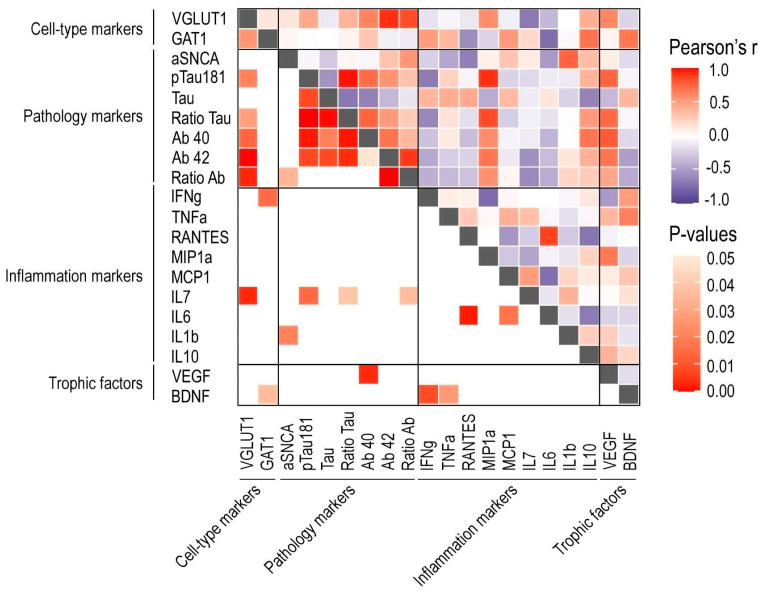
Correlation matrix for associations between VGLUT1 and GAT1 area fractions and Luminex protein concentrations in the total hippocampal area. Heat maps for *p*-values and Pearson’s r are found in the lower and upper triangles, respectively, of the matrix.

The concentrations of neuropathological, inflammatory, and neurotrophic proteins measured by Luminex were checked for correlations with VGLUT1 and GAT1 area fractions. Since protein levels were measured in homogenized hippocampi, the correlations were performed only with ISH marker data from the total hippocampus and not in a region-specific manner. A correlation matrix summarizing all associations is presented in Figure 6.

**Figure 7:**
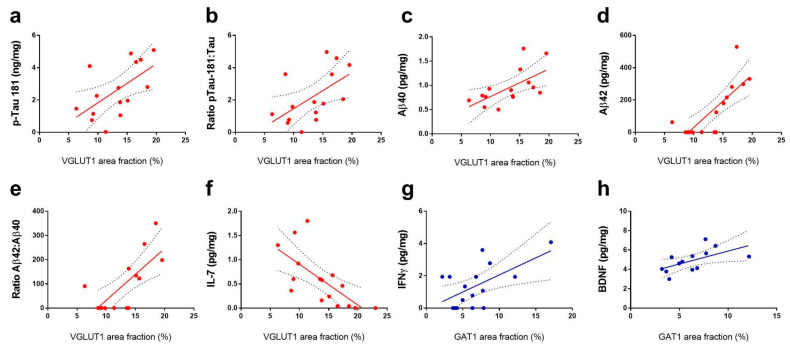
Significant correlations between VGLUT1 (red, (**a**–**f**)) and GAT1 (blue, (**g**,**h**)) area fractions and Luminex protein concentrations. Regression lines with 95% confidence intervals are shown when a significant (*p* < 0.05) relationship is observed.

Significant positive correlations were found between the VGLUT1 occupied area and the concentration of pTau-181 (r = 0.59, r^2^= 0.35, *p* = 0.02; Figure 7a) and the pTau-181 to Tau ratio (r = 0.55, r^2^= 0.31, *p* = 0.03; Figure 7b), Aβ40 (r = 0.56, r^2^= 0.31, *p* = 0.03; Figure 7c), Aβ42 (r = 0.79, r^2^= 0.62, *p* = 0.0005; Figure 7d), and Aβ42 to Aβ40 ratio (r = 0.72, r^2^= 0.54, *p* = 0.003; Figure 7e). Additionally, significant negative correlations were found between the VGLUT1 area fraction and IL-7 levels (r = −0.69, r^2^= 0.47, *p* = 0.003; Figure 7f). Lastly, significant positive correlations were found between the GAT1 area fraction and the concentrations of interferon-gamma (IFNg; r = 0.59, r^2^= 0.35, *p* = 0.01; Figure 7g) and BDNF (r = 0.58, r^2^= 0.34, *p* = 0.036; Figure 7h).

## 4. Discussion

In this study, we describe how the expression of canonical glutamatergic and GABAergic markers are influenced by age, sex, education years, and biochemical factors in the hippocampus of the nondemented elderly. Overall, we report that VGLUT1 mRNA increases in late ages are negatively correlated with IL-6 and IL-7 cytokines and positively correlated with Tau as well as Ab40 and Ab42 isoforms. The GAT1 hippocampal area is positively correlated with education years in females as well as with the concentrations of interferon-gamma and BDNF.

### 4.1. Age and Sex Effects on Glutamatergic and GABAergic Markers

We report here a higher VGLUT1 areal density in the DG and CA3/2 of non-demented individuals aged 90 years or above in comparison with donors within the 78–89-year range. A recent meta-analysis points to a number of magnetic resonance (MR) studies that show either reductions or no changes in brain glutamatergic concentrations during normal aging [31]. It is important to highlight that in 8 out of 13 of the studies analyzed by [31], the age ranges varied in a manner that the oldest subject had 78 years. The other five studies evaluated individuals with a maximum of 88 years. In a review of the recent literature, it was acknowledged that although there is a general consensus for an age-related decline in glutamatergic signaling, little information regarding the oldest-old is available [33].

It is also noteworthy that we report here VGLUT1 mRNA expression data while MR quantifies glutamate amino acid levels. A first point to consider is that VGLUT1 mRNA expression is not necessarily correlated with VGLUT1 protein levels. In a mice study [30], VGLUT1 mRNA levels decreased from young to middle age and then increased from middle age to old age in the occipital cortex. Similarly, VGLUT2 mRNA increased at old ages in the hippocampus [30]. However, both VGLUT1 and VGLUT2 protein levels decreased from young to middle age and from middle age to old age in multiple brain regions [30]. Other data from the mice showed an age-related increased VGLUT1 immunoreactivity in the cerebellum and the hypothalamus, which is accompanied by deficits in excitatory amino acid neurotransmission [48]. So, even with higher VGLUT1 protein levels, it is possible for neurons to present a lower glutamate release.

To the best of our knowledge, the present study is the first to describe how the hippocampal VGLUT1 mRNA expression changes in the octogenarian-nonagenarian range. Considering all the above-mentioned data, our findings support future investigations aiming to establish if VGLUT1 mRNA expression increases continually during aging or occurs only in later ages. Moreover, it remains to be explored if aging affects some post-transcriptional mechanism that causes reduced VGLUT1 protein and glutamate levels, despite mRNA increases. Reductions in VGLUT1 expression are documented in demented patients and are correlated with cognitive decline in this condition [49,50]. Thus, an interesting hypothesis that the present study generates is that individuals who possess age-related compensatory mechanisms for VGLUT upregulation might be less susceptible to the onset of dementia.

We found no alterations between age groups in the GAT1 area fraction. Similarly, no age-related alterations in GAT1 protein levels were observed in the sensory, motor, temporal, and cerebellar cortical areas of human brains [36]. The expression of multiple components of the GABAergic system, but not GATs, was previously quantified by Western blotting in the human hippocampus and entorhinal cortex during aging [37]. No changes in older groups were found for GAD65/67 enzymes and for the majority of the GABA receptor subunits [37]. In the visual cortex of rhesus monkeys, there were no differences in GAT1 mRNA levels between young and aged animals, although a decline can be observed from young to middle age [51]. Collectively, these data suggest that any changes in the hippocampal GABAergic neurotransmission in late ages are not likely to be related to GAT expression.

### 4.2. Associations of Biological Sex and Education Years with VGLUT1 and GAT1 Expression

No sex differences were observed between VGLUT1 or GAT1 ISH area fractions. We found no other studies regarding sex differences in VGLUT1 expression during brain aging. Notwithstanding, sex differences were documented for GABAergic components in the cortex and hippocampus of elderly individuals [36,37]. The cerebellar GAT1 protein levels are higher in older females in comparison with older males [36]. Nevertheless, no difference in GAT1 levels was found between these age groups in frontal, parietal, or temporal cortical regions [36]. The majority of GABA receptors, as well as GAD65/67 enzymes, presented no sex differences in the elderly human hippocampus [37]. However, correlations between aging and GABAAR α1, β1, β3, γ2, and GABABR R2 receptor subunits were differentially found in the hippocampal sub-regions of female and male subjects [37]. Altogether, our data contribute to the current understanding that sex differences in GABAergic neurotransmission are highly region-specific.

In our total pool of donors, no significant correlations were found between education years and VGLUT1 or GAT1 area fractions in any hippocampal subdivisions. However, when donors are grouped by sex, significant positive correlations were found between the education years and GAT1 area fraction of the DG, CA3/2, and the total hippocampus of females. No correlations with education years were found for GAT1 in males nor for VGLUT1 in both sexes.

It has been previously explored that GABAergic neurotransmission undergoes plastic changes associated with learning processes [52,53,54]. In young individuals, the hippocampal GABAergic concentration is positively correlated with retrieval performance in an associative learning paradigm [55]. Additionally, a positive correlation between hippocampal GABA concentration and episodic memory scores was found in elderly women but not in men [56]. The concept of the cognitive reserve was proposed to account for factors that might explain why some elderly individuals are more susceptible to cognitive and physiological decline while others progress in a healthier manner. Lifelong experiences, such as education, are proxies for cognitive reserve [16,57]. In this perspective, our findings may be due to education years acting as a long-term modulator of the female hippocampal GABAergic system.

It is important to highlight that the female subjects studied here are in a post-menopause stage characterized by reduced levels of ovarian hormones. It has been suggested that changes in GABA signaling are more evident in women due to the fluctuations of gonadal hormones from puberty to menopause [36,37]. Accordingly, progesterone administration can either promote or inhibit GABA receptor mRNA expression depending on the hippocampal subfield [58]. Additionally, cultured hippocampal interneurons are exposed to estradiol present and reduced GAD expression [59]. Thus, it is likely that any long-term modulatory effect that education promotes in female GABAergic neurotransmission is also mediated by these hormonal shifts.

### 4.3. Association of Neuropathological Proteins and VGLUT1 Expression

We report here that hippocampal concentrations of pTau-181 and the ratio of pTau-181 to the total Tau were positively correlated with VGLUT1 expression. This finding adds to an extensive body of evidence linking glutamatergic hyper-excitability with increasing levels of Tau [60]. For instance, TauP301L transgenic mice overexpress hyperphosphorylated isoforms of Tau, such as human pTau-181, and present a hippocampal VGLUT1 expression approximately 40% higher than wild-type strains [61]. In addition, in the dentate gyrus and in CA3, there is a significant increase in the amplitude of glutamatergic release to the extracellular medium [61]. Moreover, MR spectroscopy shows an excessive concentration of glutamate as well as the reduced conversion of glutamate to glutamine in brain homogenates from TauP301L mice [62].

Another line of evidence reinforces that mutation-induced Tau phosphorylation increases extracellular glutamate concentration and NMDA excitotoxicity in hippocampal organotypic sections [63]. In contrast, Tau knockdown in neuronal cultures decreases glutamate-mediated excitotoxicity [64]. We also corroborate a previous report of increased hippocampal activity in association with Tau levels in the inferior temporal and entorhinal cortices of cognitively normal older adults [60].

We report here a positive correlation of the VGLUT1 expression with the concentration Aβ40 and Aβ42 isoforms and with the Aβ42:Aβ40 ratio in the hippocampus of non-demented elderly individuals. Our results resemble findings in transgenic rodent strains which model the early accumulation of Aβ plaques [65,66,67]. The TgSwDI mouse strain, presents elevated levels of VGLUT1 in the hippocampus and cerebral cortex in comparison with wild-type mice [65]. In the AβPP/PS1 mouse strain, an increased hippocampal VGLUT1 expression is observed in animals submitted to either low or high-fat diets [66]. In this same model of amyloidosis, elevated glutamate levels were found to be anatomically associated with Aβ plaques in the hippocampus [67]. In hippocampal cell cultures, Aβ40 monomers mediate a complex of the amyloid precursor protein with G/i/o protein, resulting in a large presynaptic calcium flux and glutamatergic release [68]. The administration of soluble Aβ 42 was also found to promote glutamatergic release [69].

Similar to our findings, Aβ plaques preferentially accumulate nearby VGLUT1-expressing synaptosomes in the parietal cortex in AD patients [70]. There is also in vivo evidence that neurons regionally close to Aβ plaques develop a hyperactive phenotype while neurons farther away appear to be silenced [71]. Notwithstanding, Aβ plaques seem to downregulate VGLUT1 expression in patients at late stages of AD [33,72,73]. In this context, our results suggest that non-demented subjects are more likely to reach old age rather and present a positive association between Aβ and VGLUT1 than the downregulation observed in advanced AD patients.

### 4.4. IL-7 Levels Are Associated with VGLUT1 Expression

We observed a negative correlation between the hippocampal VGLUT1 area fraction and the local concentration of the inflammatory marker IL-7. IL-7 is a mediator of peripheral T cell maintenance, with a neurotrophic function and promoting neuronal and glial survival in hippocampal cell cultures [74]. In C57BL/10 mice, PCR analysis revealed that the intrathymic IL-7 concentration was decreased in older animals, and this was correlated with cognitive decline [75]. Based on our survey of the literature, the present work is the first to explore a potential relationship between IL-7 and glutamatergic activity in the elderly human hippocampus. Interestingly, we also observed significant negative correlations between the IL-7 concentration and p-Tau181 and Aβ42 concentrations. A possible scenario is that reduced IL-7 levels generate a favorable cellular environment for the formation of Aβ and Tau oligomers which are related to glutamatergic hyperactivity.

### 4.5. IFNg and BDNF Levels Are Associated with GAT1 Expression

We observed a positive correlation between GABAergic expression and IFNg concentration. IFNg regulates the activation of mononuclear phagocytes, which are secreted by activated T cells and natural Killer cells in addition to astrocytes, macrophages, and microglia [76]. IFNg increases the frequency of spontaneous inhibitory postsynaptic currents in rat hippocampal CA1 pyramidal neurons [77]. Similarly, IFNg application increased GABA-mediated synaptic inhibition in neocortical layer 5 neurons which could protect against excitotoxic events [78]. Accordingly, IFNg promotes elevated levels of GABA in the pyramidal cells of layer I/II of the prefrontal cortex of wild-type mice [79]. Thus, we corroborate here the positive relationship between IFNg levels and GABAergic neurotransmission in the hippocampus of elderly individuals.

The GAT1 area fraction was also positively correlated with BDNF concentration in the subjects analyzed here. BDNF is synthesized by neurons and glia and is responsible for providing support to neuronal growth, differentiation, and survival [80]. Higher BDNF expression is associated with slower cognitive decline in both non-demented and AD older subjects [81]. Furthermore, BNDF is one of the crucial regulators of long-term potentiation at glutamatergic and GABAergic synapses [82]. In rat hippocampal neurons, BDNF expression is positively correlated with GAD65 levels in CA1, and GABA expression selectively increases around BDNF-expressing neurons [83]. Moreover, 48 h after BDNF application in hippocampal cell cultures, the GABAergic synaptic strength and current amplitude increase [84].

In another human post-mortem study, multiple genes involved in GABAergic and glutamatergic neurotransmission were coexpressed with BDNF and downregulated with age [85]. This study further addressed this topic through a conditional blockade of BDNF/NRTK2 signaling in mice. It is reported that low BDNF levels lead to the reduction in genes involved with inhibitory, but not excitatory, neurotransmission [85]. Notwithstanding, a causal link could not be established for humans in this investigation [85]. Our findings indicate a similar effect since BDNF was correlated with GAT but not with VGLUT expression in elderly individuals. However, future investigations are still necessary to clarify cause–effect relationships.

We corroborate the above-mentioned findings that increased BDNF concentration influences greater GABAergic activity in the human hippocampus. It is also relevant to note that BDNF plays a central role in neurophysiological plasticity processes, such as memory and learning, which depend on the hippocampal function [86]. Since we also observed that older women with more education years have higher GAT1 hippocampal area fraction, future studies could investigate whether this GABAergic stimulating effect occurs through BDNF signaling.

## Figures and Tables

**Table 1:** Neuropathological, inflammatory, and growth factor proteins quantified by Luminex.

Name	Full Name	Description	Measured in
α-SNCA	Alpha Synuclein	Component of Lewy bodies neuropathological marker of Parkinson’s Disease	pg/mg
Tau	Total Tau protein	Microtubule binding protein expressed in neurons.	ng/mg
pTau-181	Tau protein phosphorylated at threonine 181	Tau abnormally phosphorylated at threonine 181: a neuropathological marker of Alzheimer’s Disease	ng/mg
pTau:Tau	Ratio of pTau-181 to Tau		
Aβ40	Beta-amyloid 1–40	40-aminoacid beta-amyloid protein, a neuropathological marker of Alzheimer’s Disease.	pg/mg
Aβ42	Beta-amyloid 1–42	42-aminoacid beta-amyloid protein, a neuropathological marker of Alzheimer’s Disease	pg/mg
Aβ42:Aβ40	Ratio of Aβ42 to Aβ40		
IFNγ	interferon-gamma	Pro-inflammatory cytokine	pg/mg
TNFα	tumor necrosis factor alpha	Pro-inflammatory cytokine	pg/mg
RANTES	Regulated on activation, normal T cells expressed and secreted	Chemokine which recruits leukocytes to inflammatory sites	pg/mg
MIP-1α	Macrophage inflammatory protein-1 alpha	Chemokine with chemotactic and pro-inflammatory effects	pg/mg
MCP-1	Monocyte chemotactic protein 1	Chemokine which recruits monocytes to inflammatory sites	pg/mg
IL-7	Interleukin-7	Growth factor which stimulates lymphocyte differentiation	pg/mg
IL-6	Interleukin-6	Pro-inflammatory cytokine	pg/mg
IL-1β	Interleukin-1β	Pro-inflammatory cytokine	pg/mg
IL10	Interleukin-10	Pro-inflammatory cytokine	pg/mg
VEGF	Vascular endothelial growth factor	Growth factor which stimulates angiogenesis	pg/mg
BDNF	Brain-derived neurotrophic factor	Neurotrophic factor which promotes neuronal growth and differentiation	pg/mg ¹

^1^ Protein quantifications and descriptions were obtained from the Aging, Dementia, and TBI study database (available at: http://aging.brain-map.org/donors/summary, accessed on 14 October 2022).

**Table 2:** Summary of donor characteristics.

Donor ID *	DSM IV	Age (Years)	Sex	Education Years	RIN	Braak	CERAD	NIA Reagan
H14.09.094	No Dementia	78	M	18	8.4	2	1	1
H14.09.074	No Dementia	82	F	16	6.7	3	1	1
H14.09.096	No Dementia	84	M	12	5.9	3	2	2
H14.09.060	No Dementia	86	F	12	5.6	4	2	2
H15.09.106	No Dementia	86	M	14	7.1	2	0	1
H14.09.078	No Dementia	87	M	16	6.5	1	0	1
H14.09.058	No Dementia	88	M	14	6.8	1	1	1
H14.09.062	No Dementia	89	F	16	7.9	3	1	1
H14.09.072	No Dementia	89	F	15	5.1	4	1	1
H14.09.090	No Dementia	89	F	12	7	5	1	2
H14.09.006	No Dementia	90–94	F	14	6.7	3	0	1
H14.09.030	No Dementia	90–94	F	15	6	3	3	2
H14.09.052	No Dementia	90–94	F	14	7.4	2	1	1
H15.09.108	No Dementia	90–94	F	12	5.5	4	3	2
H14.09.004	No Dementia	90–94	M	14	7.7	3	1	1
H14.09.102	No Dementia	95–99	F	16	7.2	1	0	1
H14.09.046	No Dementia	95–99	M	13	6.2	1	2	2
H14.09.050	No Dementia	95–99	M	8	6.7	3	3	2
H14.09.070	No Dementia	95–99	M	16	7	2	0	1

* Digital images were retrieved from the Aging, Dementia, and TBI study database (available at: http://aging.brain-map.org/donors/summary, accessed on 14 October 2022). The present study uses the same donor IDs listed in the database. DSM IV—Diagnostic and Statistical Manual of Mental Disorders 4th edition; RIN—RNA integrity numbers; BRAAK—Neurofibrillary tangle pathology scores of Braak and Braak; CERAD—Neuritic plaque pathology scores of the Consortium to Establish a Registry for Alzheimer’s Disease; NIA-Reagan—General pathology scores of the National Institute on Aging and the Ronald and Nancy Reagan Institute of the Alzheimer’s Association.

## Data Availability

Please contact the author for data requests.

## Appendix B

**Table A1:** Correlation of education years with neuronal markers in donors grouped by sex.

Hc Subfield	Correlation	VGLUT1			GAT1		
		Female	Male	Both	Female	Male	Both
DG	r	−0.13	−0.56	−0.33	0.66	0.08	0.29
	*p*	0.72	0.12	0.16	0.036 *	0.84	0.23
CA3/2	r	0.15	−0.43	−0.21	0.76	−0.3	0.07
	*p*	0.68	0.24	0.39	0.01 *	0.43	0.77
CA1	r	0.14	0.01	0.05	0.56	−0.21	0.11
	*p*	0.69	0.99	0.82	0.09	0.59	0.66
Total Hc	r	0.12	−0.26	−0.09	0.7	−0.15	0.17
	*p*	0.74	0.51	0.71	0.02 *	0.69	0.48

Hc—Hippocampus; r—Pearson’s r correlation coefficient; *p*: *p*-values. * Significant *p*-values (<0.05).
